# Harmonization of distributed multi‐center analysis based on dried blood spot reference materials supporting the screening of neonatal inherited metabolic disorders

**DOI:** 10.1002/jcla.24970

**Published:** 2023-10-13

**Authors:** Shou‐Fang Qu, Hao‐Ran Tao, Liu‐Ji Qin, Wen‐Xin Zhang, Shan Han, Shen‐Yan Zhang, Jie Huang

**Affiliations:** ^1^ Division of Diagnostic for Non‐infectious Disease National Institutes for food and drug Control (NIFDC), Institute for in Vitro Diagnostic Control Beijing China; ^2^ BGI Genomics Shenzhen China; ^3^ College of Life Sciences, University of Chinese Academy of Sciences Beijing China; ^4^ GBI Biotech, BGI Genomics Beijing China

**Keywords:** acylcarnitines, amino acids, dried blood spot, inherited metabolic disorders, reference materials, tandem mass spectrometry

## Abstract

**Background:**

The standardization of quantification data is critical for ensuring the reliability and measurement traceability in the screening of neonatal inherited metabolic disorders. However, the availability of national certified reference materials is limited in China.

**Methods:**

In this study, we developed a series of dried blood spot (DBS) reference materials containing 9 amino acids (AA) and 10 acylcarnitines (AC) for neonatal screening. Four levels of the reference materials were measured with tandem mass spectrometry (MS/MS) by seven laboratories using different commercial In Vitro Diagnostic Device (IVD) kits. Then, 100 clinical samples were measured using both derivatization and non‐derivatization methods by the same laboratory.

**Results:**

We found high homogeneity and stability at all levels of the reference materials, with the coefficient of variation (CV) of the analytes less than 15%. These reference materials can be used to assess the testing capabilities of different laboratories. Our test also revealed that the correction factors (CF) calculated by the reference materials, along with clinical samples, could increase the consistency for different kits.

**Conclusion:**

The DBS reference materials proposed in this study provide reliability for the harmonization in multi‐center analysis for the screening of neonatal inherited metabolic disorders. And applying our correction method for the screening could improve the data consistency of the DBS samples prepared by different methods.

## Graphical abstract

The flowchart outlines our research process. A range of evaluations were carried out after the development of DBS reference materials, which are applicable to correct the results of diverse laboratories, kits and platforms. We anticipate our products will ensure dependable harmonization and standardization for multi‐center metabolism analysis in the future.

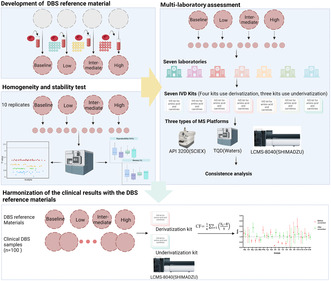

## 1 INTRODUCTION

Inherited metabolic disorders are genetic conditions resulting in metabolism problems, and several treatment strategies have been developed after diagnosis. The early screening, diagnosis, and therapeutic intervention is critical for several disorders, such as Phenylketonuria (PKU)^1^ and Congenital hypothyroidism (CH).^2^ Compared with biochemical assays and radioimmunoassay that were relatively laborious, neonatal screening based on tandem mass spectrometry (MS/MS) has advantages in specificity, and could quantify multiple metabolites by one injection.^3^, ^4^ The main sample type for this screening is DBS, which is beneficial for collecting little sample volume, with easily transported and storage.^5^ This less‐invasive and economic blood collection method, however, is sensitive to blood conditions and manufacturing conditions,^6^, ^7^ and slight differences of operation can lead to totally different results. So, ensuring the sample homogeneity of DBS is more than important.

The sample preparation methods of DBS for neonatal screening include derivatization and non‐derivatization techniques.^8^, ^9^, ^10^ For low‐sensitive mass spectrometers, derivatization method has been introduced to improve the signals detected by MS, which uses the principle that n‐butanol can react with the carboxyl groups of amino acids or acylcarnitines to form butyl ester derivatives to increase the efficiency of ionization.^9^ While for mass spectrometers with higher sensitivity, the non‐derivatization methods have attracted increasing attention.^11^ Samples could be injected for MS analysis directly after extraction without derivatization,^11^, ^12^ avoiding potential hazards to the environment and human health by derivatization reagents and minimizing specimen manipulation. In China, only a few commercial kits have been approved by the National Medical Products Administration of China (NMPA) including both derivatization and non‐derivatization methods. Besides, several MS instruments developed by different manufacturers have been approved for the screening of neonatal inherited metabolic disorders. Although all kits' manufacturers performed meteorological tracing according to ISO17511 as reported,^13^ there are differences among the clinical reference ranges of each analyte according to the manuals in those kits,^14^ which indicate that there is still a need for national evaluation across laboratories in China based on certified reference materials.

The standardization of quantification data is critical to the reliability and measurement traceability in the screening of neonatal inherited metabolic disorders,^15^, ^16^, ^17^ and developing DBS reference materials is the base step in such terms. A set of DBS reference materials with great traceability can play a significant role in DBS‐based screening, providing secondary calibration for specific measuring systems,^18^ which is very limited across the world. Moreover, most neonatal screening reference materials contain only a few kinds of amino acids,^18^, ^19^ and cannot fully reflect the metabolic disorders of the newborns.

Besides, considering the derivatization and non‐derivatization methods rely on semi‐quantitative assessments for the diagnosis of metabolic diseases, based on the ratio of the peak intensity of native metabolites and their isotope‐labeled counterpart.^8^, ^9^, ^10^ Herein, we developed a series of dried blood spot (DBS) reference materials containing 9 amino acids (AA) and 10 acylcarnitines (AC) for neonatal screening, representative of the most common inherited AA and AC metabolic disorders in newborn screening. Based on these reference materials, we distributed them to multi‐centers and evaluated the standardization in 100 clinical samples with both derivatization and non‐derivatization methods.

## 2 MATERIALS AND METHODS

### 2.1 Materials

Free carnitine (C0), acetylcarnitine (C2), butyl carnitine (C4), octyl carnitine (C8), and kwai carnitine (C10) were purchased from Toronto Research Chemicals. Propionylcarnitine (C3), hexanoylcarnitine (C6), lauroyl carnitine (C12), myristoyl carnitine (C14), and palmitoyl carnitine (C16) were purchased from Chem‐Impex International Inc. Alanine (Ala), valine (Val), citrulline (Cit), leucine (Leu), methionine (Met), phenylalanine (Phe), proline (Pro), and tyrosine (Tyr) were purchased from Sigma. Glycine (Gly) was purchased from the National Institutes for Food and Drug Control. Methanol and acetonitrile of HPLC‐MS grade were purchased from Fisher Scientific. The filter paper was from Whatman. Plasma was purchased from Scantibodies Laboratory Inc. (product number: 3SH027, batch number: M475D). Red blood cells were separated from the blood of an anonymous volunteer donor tested negative for HIV, TP, HBV, and HCV.

### 2.2 Preparation of the DBS reference material

The red blood cells were rinsed six times with saline. An equal volume of plasma was added, mixed with the cells, and used as the baseline of whole blood. The solutions that contained three different concentration levels of 9 amino acids and 10 acylcarnitines (Table S1) were added to obtain three preparations of the whole baseline blood. Then, 50 μL each of the four preparations of whole blood (marked as baseline, low, intermediate, and high levels) were used to make DBSs on Whatman 903 filter papers. The DBSs were dried at room temperature for 3–4 h and then vacuum‐packed in aluminum foil bags along with silica‐gel desiccants. Finally, those DBS reference materials were stored at −70°C before use or shipping.

### 2.3 Homogeneity test

Homogeneity testing is of great importance for the certification of reference materials, as it could inspect the validity of the certified values and their uncertainties in this previous analysis.^20^ The homogeneity test was performed according to the requirements described in the ISO Guide 35,^21^ with 10 sets of randomly selected DBS reference materials tested by MS. Three dots from each DBS were measured (the diameters of the dots were about 3.2 mm). The *F*‐test was performed for each DBS level, and the coefficient of variation (CV) was calculated.

### 2.4 Stability test

In newborn DBS screening, the timeliness of the sample should also be given priority.^22^ Since specimens can be shipped nationwide within 7 days in China, the stability of the DBS reference materials was assessed at three different temperatures: −20°C, 2–8°C, and 37°C, and after 0, 1, 2, 3, 5, and 7 days of storage. Unitary linear regression analysis was performed using Microsoft Excel 2016. The *t*‐test was performed according to the requirements described in the ISO Guide 35^21^ to verify the shipping stabilities of the reference materials at different temperatures.

### 2.5 Evaluation of the DBS results in seven different laboratories with multiple IVD kits and multiple MS platforms

Ten series of DBS references were distributed to seven laboratories (Labeled as Lab A, B, C, D, E, F, and G). For each DBS, three blood dots were collected sequentially, and each dot was measured with three replicates for MS analysis. All laboratories performed the sample preparation and consequent analyses using the commercial IVD kits and MS instruments registered and approved by the NMPA (the information of the kits and instruments used in the laboratories are shown in Table S2). The non‐derivatization method was used by laboratories A, B, C, D, and E, while the derivatization method was used by laboratories B, C, F, and G. The repeatability and extraction recovery of the reference materials reported by the laboratories were assessed. To calculate the extraction recovery, the test results of the low‐, intermediate‐, and high‐level reference materials were subtracted by the value of the baseline reference material, and divided by the concentrations of analytes added.

### 2.6 Harmonization of the clinical results with the DBS reference materials

After obtaining informed consent, the leftover DBSs from 100 newborns collected at 3–22 days after delivery and tested at laboratory G were used for this evaluation. Two dots in their DBS were tested for each newborn. The blood dots were tested by the derivatization (using the kits F–I) and non‐derivatization (using the kits B–II) methods. All 100 clinical samples and the four levels of reference materials were prepared simultaneously and then analyzed. The correction factor (CF) of each analyte was calculated according to the DBS reference materials, using the equation described below. Then, the measured concentration of each analyte was divided by the corresponding CF to obtain the corrected concentration. If the corrected concentration of the analyte in the reference material was within the range of the theoretical concentration ± 20%, the CF of the corresponding analyte was considered valid and could be used to correct the results of the clinical sample. Afterward, the Bland–Altman method, a simple parametric approach proposed based on analysis of variance and simple graphical methods, was used to test the consistency of clinical samples' results, measured by both the derivatization and non‐derivatization methods, before and after correction.^23^ The changes in the consistency among the tested results before and after correction were evaluated.

The equation for calculating the CF of the analytes was

$$\mathrm{CF}=\frac{1}{n}\sum_{i=1}^{n}\left(\frac{X_{i}-B}{Y}\right)$$

where CF is the correction factor of the corresponding analyte, *n* is the number of valid measurements of the analyte, *X*_*i*_ is the tested results of the analyte in reference materials of different levels, *B* is the tested result of the analyte in the baseline reference material, and *Y* is the concentration of the tested analyte added to the baseline reference materials.

## 3 RESULTS

### 3.1 Homogeneity evaluation based on developed DBS reference materials

**FIGURE 1:**
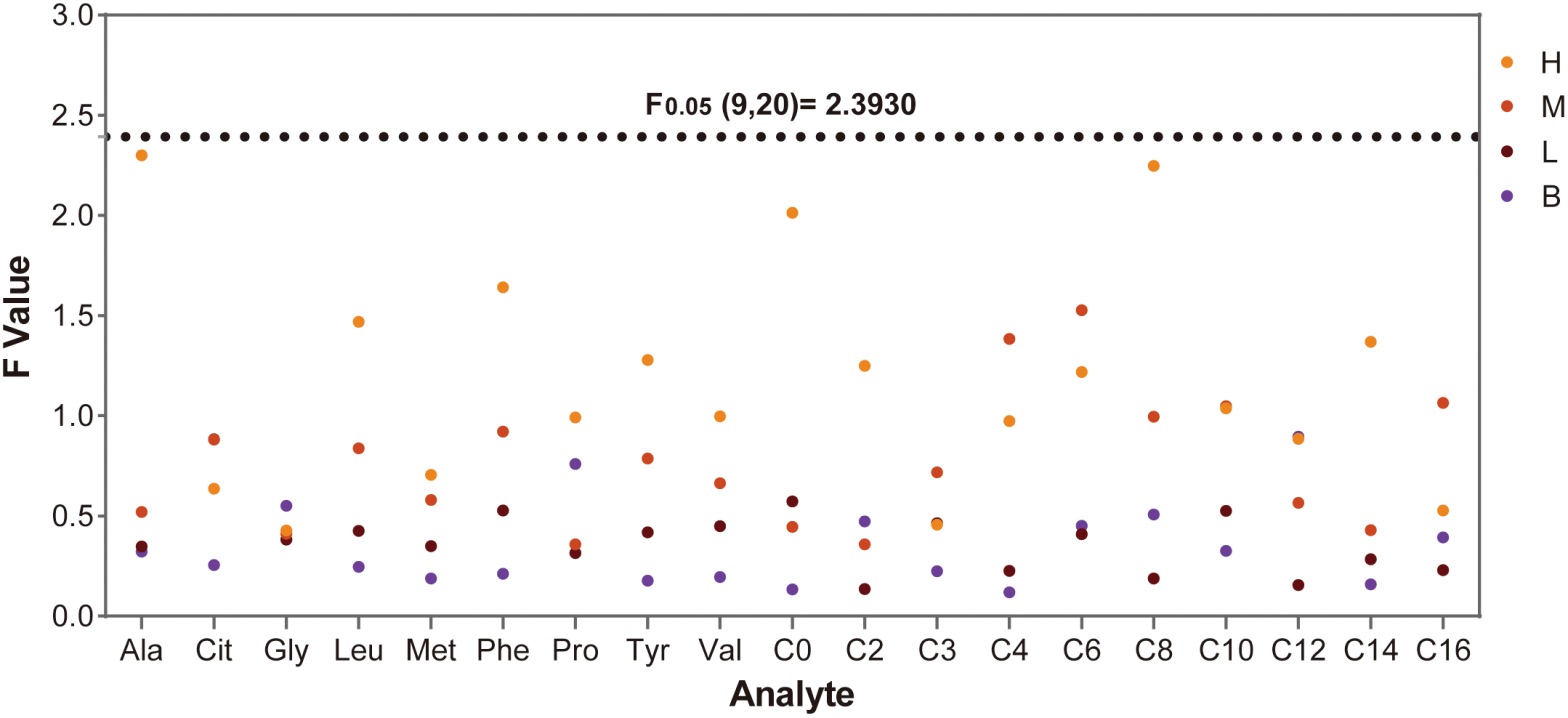
Homogeneity test of the DBS reference materials. H, M, L, and B stand for the homogeneity of each reference compound in the high‐level, intermediate‐level, low‐level, and baseline reference material. The *F* values of all the results were below *F*_0.05_(9,20).

For the DBS reference materials of each level, 10 DBSs were randomly selected. For each DBS, three blood dots were cut for the test. One‐way analysis of variances (ANOVA) was performed for the tested results, and the CVs of each analyte in the reference materials of different levels were calculated. The ANOVA results showed that the *F*‐values of the tested results of all the analytes were below *F*_0.05_(9,20), suggesting that there was no significant difference among those DBSs, and the DBSs of each level prepared in this study met the criteria of homogeneity. In addition, the CVs of the analytes above their limits of detection were less than 10% (average CV was 3.64%). The *F* values of the reference materials of the four levels are shown in Figure 1. The inter‐DBS CVs are shown in Table 1.

**TABLE 1:** CVs of the substances in the reference materials of the four levels (*n* = 30).

Analyte	Blank, %	Low, %	Mid, %	High, %
Ala	1.92	2.30	2.56	2.94
Cit	4.39	4.55	3.28	3.37
Gly	2.19	2.59	3.02	3.98
Leu	1.68	2.39	3.21	3.07
Met	4.36	2.98	4.62	3.84
Phe	1.74	2.74	2.73	2.97
Pro	4.67	3.49	4.89	4.91
Tyr	1.74	2.08	2.95	2.76
Val	2.00	2.75	3.09	2.69
C0	1.88	3.15	2.99	4.36
C2	4.03	2.49	3.80	5.16
C3	2.54	3.93	4.51	3.42
C4	5.71	3.43	4.28	4.37
C6	—^a^	4.43	5.00	5.20
C8	— ^a^	3.64	5.74	7.23
C10	—^a^	4.91	5.71	6.24
C12	—^a^	2.38	3.87	3.11
C14	—^a^	3.08	3.93	4.10
C16	3.43	2.45	4.45	3.72

^a^ The concentrations of C6, C18, C10, C12, and C14 in the baseline reference material were lower than the lower limit of detection.

### 3.2 Stability assessment of DBS reference materials

Following the certification procedure, 7‐days stability for storage conditions were confirmed. The linear fitting method was used for the 7‐day stability test of the reference materials at three different temperatures. The *t*‐test showed that the slopes of all analytes in the DBS reference materials ($\left|b_{1}\right|$) were all <$t_{0.95,4}•s\left(b_{1}\right)$, suggesting that the vacuum‐packed DBS reference materials could be stably preserved for 7 days at temperatures of −20°C, 2–8°C, and 37°C (Tables S3–S5). So those sealed DBS materials were able to transfer to other laboratories within 7 days in dry ice without compromising their integrity. In addition, the DBS reference materials were also proved stable when preserved at 37°C for 7 days, suggesting that the actual storage time of the reference materials at low temperature (4°C) could be about half a year, according to the Arrhenius equation.

### 3.3 Comparative study of the DBS reference materials in seven laboratories

**FIGURE 2:**
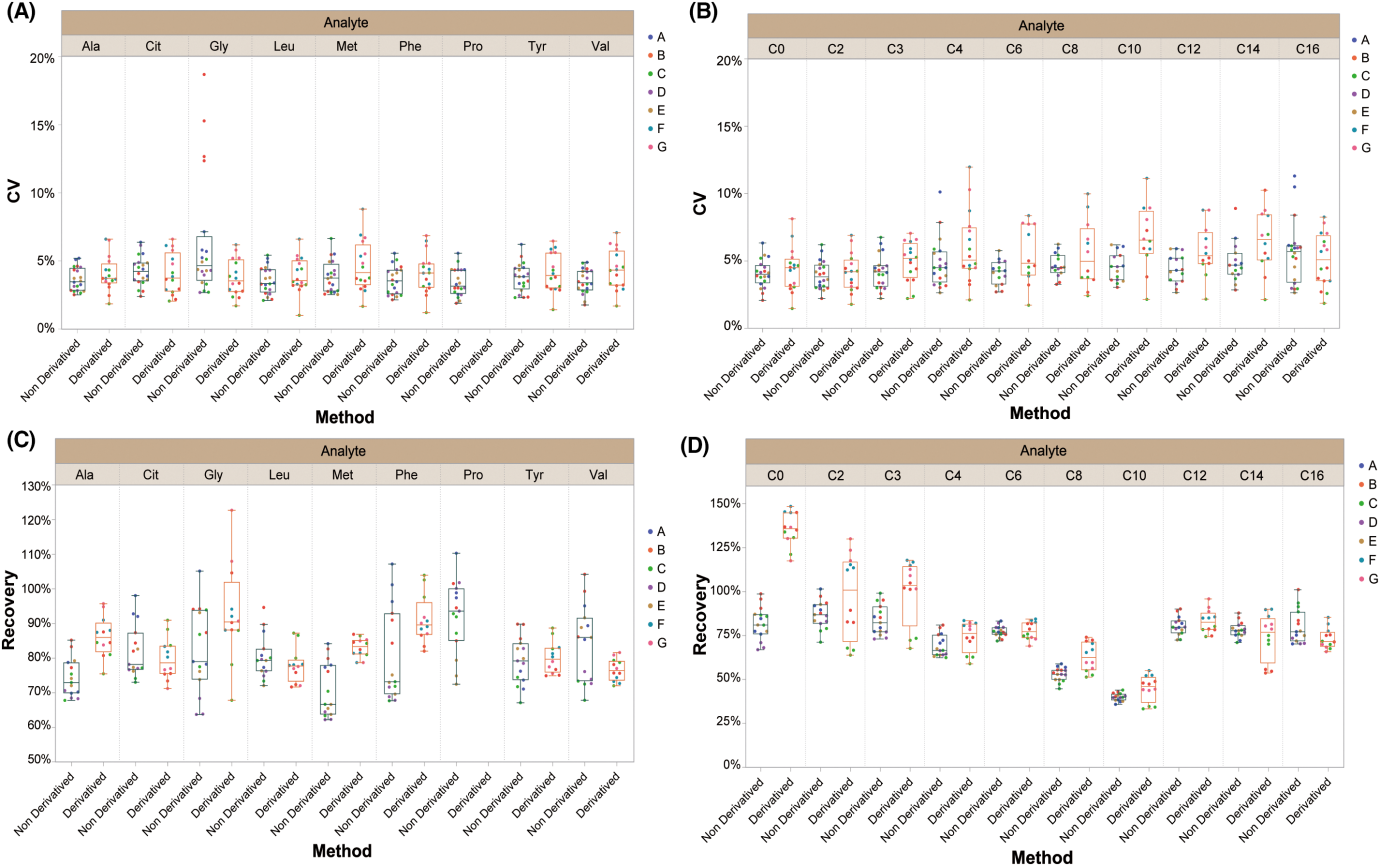
Repeatability of analytes and the extraction recovery rates of the DBS reference materials across the seven laboratories (Table S2). (A) Repeatability distribution of quantified amino acids. (B) Repeatability distribution of quantified acylcarnitines. (C) Extraction recovery rate of amino acids. (D) Extraction recovery rate of acylcarnitines. The boxplots show the distribution of the extraction recovery rates in the laboratories, labeled with the Max, Q3, Med, Q1, and Min of the tested results, respectively.

Seven laboratories participated in the assessment of the DBS reference materials, of which five laboratories used non‐derivatization method on two types of MS platforms (including two laboratories using the SCIEX API3200MD and three laboratories using Waters Xevo TQD), and four laboratories used derivatization method on three types of MS platforms (including one API3200MD, two Waters Xevo TQD, and one SHIMADZU LCMS‐8040). All instruments and reagents used in these laboratories were approved by the NMPA. The CVs and extraction recovery rates of the reference materials with four levels were tested in the seven laboratories. Although the repeatability varied among the laboratories, the CVs of each analyte in most laboratories were less than 10% (Figure 2A,B). The recovery rate for most of the 9 amino acids and 10 acylcarnitines in the laboratories could not reach to 100% no matter which kits were used (Figure 2C,D), except for testing some short‐chain acylcarnitines by the derivatization method (due to side reactions, the extraction recovery rate for the short‐chain carnitines should be above 100% compared with added concentrations of analytes).

### 3.4 Harmonization of the clinical results with the DBS reference materials

**FIGURE 3:**
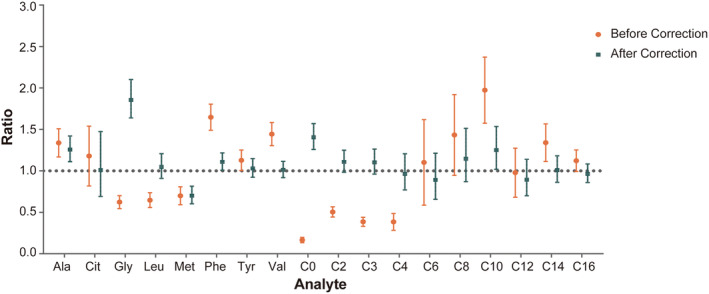
Ratio of the test results of clinical samples before and after the CF correction. Ratio: the ratio of the non‐derivatization result to derivatization result of each sample. Figure 3 shows the mean value of the 100 samples, and the error bars show the 1.96 SD.

In order to evaluate the harmonization in clinical application, 100 clinical DBS samples were prepared and quantified using the derivatization and non‐derivatization methods along with a set of DBS reference materials. Since the corrected concentrations of the analytes in the DBS materials were within the acceptable range (±20%) of their theoretical concentration, the CF of the corresponding analyte was considered valid and could be used to correct the results of the clinical sample. The Bland–Altman method was adopted to assess the consistency between the results before and after the CF correction from the derivatization and non‐derivatization methods. The results from the tests with different sample preparation methods are shown in Figure 3. The mean ratios of most analytes exceed the reference range of 0.9–1.1 before correction, except C12, suggesting that the consistency between the two methods was relatively poor. Nevertheless, after correction by the CF, the consistency of all the analytes, except for Gly, increased substantially, and the mean ratio of Cit, Leu, Tyr, Val, C4, C6, C12, C14, and C16 were within the range of 0.9–1.1. It means that the consistency between two different methods signified a significant improvement. Therefore, the DBS reference materials can prepare and test along with clinical samples in the future. The four levels of the reference materials enable the calculation of a lab‐specific CF value in the coprocessing. Such CF‐corrected data would be more consistent among intra‐ or even inter‐lab tests. Applying such DBS reference materials in clinical examinations might promote the reciprocal recognition of the testing results from different hospitals.

## 4 DISCUSSION

The national screening of neonatal inherited metabolic disorders in dried blood spots (DBS) requires standardization and harmonization across multiple laboratories. In this study, we proposed a series of DBS reference materials with four concentration levels covering the detection range of 9 amino acids (AA) and 10 acylcarnitines (AC) in China. The preparation and testing of the reference materials were done according to applicable guidelines and principles,^21^ indicating that they could be used for standardized clinical measurements. System evaluation was applied to the series of DBS reference materials, revealing their high homogeneity and good stability. Since the present study did not examine durations longer than 7 days, on the account that specimens can be shipped country‐wide within this schedule. Nevertheless, more studies are needed to investigate the long‐term stability of the DBS reference materials in the future.^24^

In general, the derivatization method involved more sample preparation procedures and might introduce more operational errors. Therefore, the repeatability of the derivatization method in the laboratories was relatively lower than the non‐derivatization method, especially in the quantification of acylcarnitines. Glycine has a very small molecular weight and could be easily influenced by background noise during MS tests, which could decrease the test accuracy, as previously observed. Nevertheless, the molecular weight of glycine increased after derivatization, and thus the stability of the derivatization method was higher than the non‐derivatization method when testing glycine. As the derivatization kits we used did not include proline for measurement, no proline results in the clinical samples before and after correction were reported in this study. Although the repeatability of different laboratories varied in this study, the repeatability among different MS instruments was not significantly different.

This study has limitations since only the kits and MS/MS systems approved for neonatal screening by the NMPA were tested. However, the reference materials prepared in this study were proven to have high homogeneity and stability and could be used to assess the testing capabilities of different laboratories. Applying the correction method for the screening of clinical samples could improve the consistency of different measurement methods currently used in clinical laboratories. In the future, if new metabolic biomarkers in DBS are found, their reference material could also be prepared, verified, and applied in clinical practices according to the methods reported in this study.

## AUTHOR CONTRIBUTIONS

Conceptualization and study design, S.‐Y. Z., and J. H.; methodology, S.‐F. Q., L.‐J. Q., and W.‐X. Z.; data collection, S.‐F. Q., L.‐J. Q., W.‐X. Z., and S. H.; statistical analysis, S.‐F. Q. and H.‐R. T.; data curation, S. H. and B. L.; writing, S.‐F. Q. and H.‐R. T.; critical revision of the manuscript, H.‐R. T. and S.‐Y. Z.; study supervision, J. H. All authors have read and agreed to the published version of the manuscript.

## CONFLICT OF INTEREST STATEMENT

The authors declare that they have no conflict of interest.

## Supporting information


Table S1–S5.

## Data Availability

The data sets supporting the results of this article are included within the article. The data sets used and/or analyzed during the current study are available from the corresponding author on reasonable request.
