# Supplementary material for: Harmonization of distributed multi‐center analysis based on dried blood spot reference materials supporting the screening of neonatal inherited metabolic disorders
Source: J Clin Lab Anal. 2023 Oct 13;37(19-20):e24970. doi: 10.1002/jcla.24970 (PMC10681404; doi:10.1002/jcla.24970)
Supplement: Supplementary file 1 — Table S1–S5. [file JCLA-37-e24970-s001.docx]

Standardization and harmonization of distributed multi-center analysis based on dried blood spot reference materials supporting the screening of neonatal inherited metabolic disorders

Shou-Fang Qu PhD^1, †^, Hao-Ran Tao BS^3,4, †^, Liu-Ji Qin BS^3^, Wen-Xin Zhang PhD^1^, Shan Han MS^2^, Shen-Yan Zhang PhD^2,3,^ *, Jie Huang PhD^1,^ *

^1^ National Institutes for food and drug Control (NIFDC), Institute for in Vitro Diagnostic Control, division of Diagnostic for Non-infectious Disease, Beijing, China.

^2^ Beijing BGI-GBI Biotech Co., Ltd, Beijing, China.

^3^ BGI Genomics, Shenzhen, China.

^4^ University of the Chinese Academy of Sciences, Beijing, China

***** Author to whom correspondence should be addressed.

**†** These authors contributed equally to this work.

**Supplementary Table S1.** Concentrations of the substances added to the three levels of reference standards (μmol/L)

| **Substance** | **Low-level** | **Intermediate-level** | **High-level** |
| --- | --- | --- | --- |
| C0 | 9.78 | 19.57 | 39.13 |
| C2 | 9.80 | 19.60 | 39.20 |
| C3 | 2.94 | 5.88 | 11.76 |
| C4 | 0.95 | 1.89 | 3.79 |
| C6 | 0.49 | 0.99 | 1.97 |
| C8 | 0.44 | 0.87 | 1.74 |
| C10 | 0.49 | 0.98 | 1.96 |
| C12 | 0.99 | 1.98 | 3.96 |
| C14 | 0.49 | 0.99 | 1.98 |
| C16 | 2.97 | 5.94 | 11.88 |
| Ala | 197.82 | 395.64 | 791.29 |
| Cit | 50.00 | 100.01 | 200.01 |
| Gly | 200.00 | 400.00 | 800.00 |
| Leu | 100.18 | 200.37 | 400.74 |
| Met | 50.11 | 100.23 | 200.45 |
| Phe | 98.95 | 197.89 | 395.78 |
| Pro | 100.01 | 200.01 | 400.03 |
| Tyr | 199.17 | 398.35 | 796.69 |
| Val | 200.04 | 400.09 | 800.17 |

***** C0: pure free carnitine; C2: acetylcarnitine; C4: butyl carnitine; C8: octyl carnitine; C10: kwai carnitine; C3: propiylcarnitine; C6: hexanoylcarinitine; C12: lauroyl carnitine; C14: myristoyl carnitine; C16: palmitoyl carnitine; ala: alanine; Val: valine; Cit: citrulline; Leu: leucine; Met: methionine; Phe: phenylalanine; Pro: proline; Tyr: tyrosine; Gly: glycine.

**Supplementary Table S2.** Information about the kits and instruments used in the laboratories

| Laboratory | Code of kit | Name of kit | Kit manufacturer | Equipment |
| --- | --- | --- | --- | --- |
| **A** | Non-derivatization (A-II) | Non-derivatization kit for screening of multiple neonatal inherited metabolic diseases (MS/MS) | Darui Biotechnology Co., Ltd | API3200MD |
| **B** | Derivatization (B-I) | Derivatization kit for multiple amino acids and carnitines (MS/MS) | Guangzhou Fenghua Biological Engineering Co., Ltd | API3200MD |
|  | Non-derivatization (B-II) | Kit for testing succinylacetone and non-derivatization measurement of multiple amino acids and carnitines (MS/MS) | Guangzhou Fenghua Biological Engineering Co., Ltd | API3200MD |
| **C** | Non-derivatization (C-II) | Non-derivatization kit for multiple amino acids, carnitines, and succinylacetone (MS/MS) | PerkinElmer | Waters Xevo TQD |
|  | Derivatization (C-I) | Kit for multiple amino acids and carnitines (MS/MS) | PerkinElmer | Waters Xevo TQD |
| **D** | Non-derivatization (D-II) | Non-derivatization kit for multiple neonatal inherited metabolic diseases (MS/MS) | PerkinElmer | Waters Xevo TQD |
| **E** | Non-derivatization (C-II) | Non-derivatization kit for multiple amino acids, carnitines, and succinylacetone (MS/MS) | PerkinElmer | Waters Xevo TQD |
| **F** | Derivatization (F-I) | Kit for amino acids and carnitines (LC-MS/MS) | BGI Biotechnology (Wuhan) Co., Ltd | Waters Xevo TQD |
| **G** | Non-derivatization (B-II) | Kit for testing succinylacetone and non-derivatization measurement of multiple amino acids and carnitines (MS/MS) | Guangzhou Fenghua Biological Engineering Co., Ltd | LCMS-8040 |
| **G** | Derivatization (F-I) | Kit for amino acids and carnitines (LC-MS/MS) | BGI Biotechnology (Wuhan) Co., Ltd | LCMS-8040 |

***** MS: mass spectrometry; LC: liquid chromatography.

**Supplementary Table S3.** Stability of the standards in dried blood spot reference standards of 4 levels within 7 days at -20°C

| **Analyte** | **Baseline** | | | **Low** | | | **Intermediate** | | | **High** | |
| --- | --- | --- | --- | --- | --- | --- | --- | --- | --- | --- | --- |
|  | \|b1\| | sb1*t (0.95,n-2) | \|b1\| | | sb1*t (0.95,n-2) | \|b1\| | | sb1*t (0.95,n-2) | | \|b1\| | sb1*t (0.95,n-2) |
| Ala | 0.9787 | 5.8949 | 1.1737 | | 5.3310 | 5.6410 | | | 8.8899 | 2.6877 | 11.6926 |
| Cit | 0.2457 | 0.6260 | 0.1553 | | 1.1846 | 2.4400 | | | 2.5902 | 2.5533 | 4.2677 |
| Gly | 3.6464 | 7.0983 | 7.1104 | | 14.8440 | 2.7957 | | | 10.7741 | 5.7479 | 11.2554 |
| Leu | 0.1644 | 0.5605 | 1.0817 | | 1.8607 | 1.6708 | | | 2.0196 | 1.8762 | 2.8413 |
| Met | 0.2308 | 0.4552 | 0.1623 | | 0.9479 | 1.0032 | | | 1.0427 | 0.7541 | 2.4192 |
| Orn | 0.2587 | 1.0151 | 1.7398 | | 3.2726 | 4.6442 | | | 37.6335 | 3.5023 | 5.7587 |
| Phe | 0.4075 | 0.7162 | 0.7056 | | 1.3477 | 1.7758 | | | 1.9906 | 1.0497 | 3.1019 |
| Tyr | 0.3132 | 0.5476 | 1.8766 | | 92.8168 | 2.6474 | | | 5.2911 | 6.3398 | 16.9156 |
| Val | 0.0063 | 0.5900 | 0.2377 | | 1.6894 | 1.1396 | | | 2.1785 | 0.1774 | 3.6397 |
| C0 | 0.0590 | 0.0832 | 0.0658 | | 0.0831 | 0.0710 | | | 0.0858 | 0.0949 | 0.1404 |
| C2 | 0.0106 | 0.0884 | 0.1086 | | 0.3770 | 0.0934 | | | 0.1246 | 0.0497 | 0.6028 |
| C3 | 0.0035 | 0.0119 | 0.0202 | | 0.0328 | 0.0355 | | | 0.0604 | 0.0023 | 0.0653 |
| C4 | 0.0019 | 0.0027 | 0.0027 | | 0.0065 | 0.0046 | | | 0.0093 | 0.0087 | 0.0155 |
| C6 | 0.0007 | 0.0021 | 0.0044 | | 0.0114 | 0.0139 | | | 0.0145 | 0.0017 | 0.0378 |
| C8 | 0.0025 | 0.0064 | 0.0045 | | 0.0104 | 0.0012 | | | 0.0126 | 0.0004 | 0.0229 |
| C10 | 0.0021 | 0.0024 | 0.0067 | | 0.0150 | 0.0036 | | | 0.0282 | 0.0118 | 0.0286 |
| C12 | 0.0003 | 0.0005 | 0.0067 | | 0.0376 | 0.0074 | | | 0.0099 | 0.0067 | 0.0241 |
| C14 | 0.0018 | 0.0031 | 0.0031 | | 0.0070 | 0.0074 | | | 0.0099 | 0.0008 | 0.0138 |
| C16 | 0.0003 | 0.2761 | 0.0599 | | 0.2871 | 0.0264 | | | 0.0751 | 0.0297 | 0.0891 |

***** Ala: alanine; Val: valine; Cit: citrulline; Leu: leucine; Met: methionine; Phe: phenylalanine; Pro: proline; Tyr: tyrosine; Gly: glycine; C0: pure free carnitine; C2: acetylcarnitine; C4: butyl carnitine; C8: octyl carnitine; C10: kwai carnitine; C3: propiylcarnitine; C6: hexanoylcarinitine; C12: lauroyl carnitine; C14: myristoyl carnitine; C16: palmitoyl carnitine.

**Supplementary Table S4.** Stability of the substances in dried blood spot reference standards of 4 levels within 7 days at 4°C

| Analyte | Baseline | | | Low | | | Intermediate | | | High | | |
| --- | --- | --- | --- | --- | --- | --- | --- | --- | --- | --- | --- | --- |
|  | \|b1\| | sb1*t (0.95,n-2) | \|b1\| | | sb1*t (0.95,n-2) | \|b1\| | | sb1*t (0.95,n-2) | \|b1\| | | sb1*t (0.95,n-2) |  |
| Ala | 2.1345 | 6.8800 | 0.4578 | | 10.9532 | 2.2237 | | 14.9017 | 3.2111 | | 22.3875 |  |
| Cit | 0.4752 | 1.6602 | 0.0451 | | 1.4421 | 0.9811 | | 2.5522 | 2.0521 | | 7.5004 |  |
| Gly | 9.6047 | 11.6678 | 4.8187 | | 7.4072 | 0.0372 | | 18.1231 | 2.0350 | | 27.2666 |  |
| Leu | 0.5804 | 0.6661 | 0.5490 | | 2.0110 | 0.9047 | | 4.5423 | 0.5900 | | 8.7543 |  |
| Met | 0.2393 | 0.5110 | 0.1071 | | 0.8604 | 1.0241 | | 3.0136 | 0.0042 | | 5.1718 |  |
| Orn | 0.7404 | 1.0285 | 1.4842 | | 3.3730 | 1.7966 | | 2.6090 | 3.5015 | | 9.5250 |  |
| Phe | 0.1806 | 0.4219 | 0.4044 | | 1.5651 | 0.5931 | | 6.3569 | 0.7591 | | 15.5993 |  |
| Tyr | 0.4347 | 0.8784 | 0.7170 | | 4.0577 | 0.2653 | | 7.7657 | 0.7533 | | 28.3895 |  |
| Val | 0.3275 | 0.4664 | 0.5500 | | 1.8607 | 1.2214 | | 6.0930 | 2.3638 | | 15.1974 |  |
| C0 | 0.0725 | 0.0901 | 0.0804 | | 0.1181 | 0.0119 | | 0.3163 | 0.0072 | | 0.5369 |  |
| C2 | 0.1189 | 0.1503 | 0.0869 | | 0.2363 | 0.1838 | | 0.3576 | 0.0660 | | 1.0425 |  |
| C3 | 0.0136 | 0.0186 | 0.0022 | | 0.0600 | 0.0148 | | 0.0915 | 0.0190 | | 0.2059 |  |
| C4 | 0.0026 | 0.0035 | 0.0016 | | 0.0100 | 0.0023 | | 0.0277 | 0.0023 | | 0.0460 |  |
| C6 | 0.0013 | 0.0112 | 0.0060 | | 0.0070 | 0.0092 | | 0.0419 | 0.0154 | | 0.0616 |  |
| C8 | 0.0005 | 0.0035 | 0.0030 | | 0.0137 | 0.0020 | | 0.0298 | 0.0069 | | 0.0492 |  |
| C10 | 0.0032 | 0.0043 | 0.0016 | | 0.0118 | 0.0085 | | 0.0271 | 0.0051 | | 0.0742 |  |
| C12 | 0.0012 | 0.0052 | 0.0051 | | 0.0078 | 0.0039 | | 0.0261 | 0.0048 | | 0.0915 |  |
| C14 | 0.0019 | 0.0057 | 0.0017 | | 0.0061 | 0.0001 | | 0.0172 | 0.0054 | | 0.0647 |  |
| C16 | 0.0128 | 0.0261 | 0.0020 | | 0.0518 | 0.0013 | | 0.2595 | 0.0169 | | 0.5197 |  |

***** Ala: alanine; Val: valine; Cit: citrulline; Leu: leucine; Met: methionine; Phe: phenylalanine; Pro: proline; Tyr: tyrosine; Gly: glycine; C0: pure free carnitine; C2: acetylcarnitine; C4: butyl carnitine; C8: octyl carnitine; C10: kwai carnitine; C3: propiylcarnitine; C6: hexanoylcarinitine; C12: lauroyl carnitine; C14: myristoyl carnitine; C16: palmitoyl carnitine.

**Supplementary Table S5.** Stability of the substances in dried blood spot reference standards of 4 levels within 7 days at 37°C

| **Analyte** | **Baseline** | | **Low** | | | **Intermediate** | | | **High** | |
| --- | --- | --- | --- | --- | --- | --- | --- | --- | --- | --- |
|  | \|b1\| | sb1*t (0.95,n-2) | \|b1\| | sb1*t (0.95,n-2) | \|b1\| | | sb1*t (0.95,n-2) | \|b1\| | | sb1*t (0.95,n-2) |
| Ala | 2.1329 | 4.0946 | 2.1698 | 10.0554 | 7.6123 | | 13.5264 | 3.5561 | | 13.6147 |
| Cit | 0.2930 | 0.3539 | 1.4532 | 1.9654 | 0.8471 | | 2.6192 | 3.3714 | | 3.4231 |
| Gly | 0.6676 | 12.7380 | 2.6748 | 16.4769 | 7.6640 | | 11.4622 | 3.3473 | | 14.1513 |
| Leu | 0.1977 | 0.7901 | 0.7132 | 3.2481 | 1.4002 | | 4.2868 | 1.5724 | | 5.0448 |
| Met | 0.1913 | 0.4357 | 0.6318 | 0.8334 | 1.3620 | | 1.5104 | 0.4830 | | 0.6962 |
| Orn | 0.0401 | 1.0563 | 1.3907 | 4.2714 | 2.7777 | | 9.2081 | 6.0847 | | 6.2985 |
| Phe | 0.1102 | 0.6234 | 0.0622 | 2.4394 | 2.3407 | | 5.0366 | 0.1275 | | 4.8996 |
| Tyr | 0.2629 | 0.5213 | 1.2496 | 5.2167 | 5.8291 | | 11.7438 | 0.8466 | | 15.3259 |
| Val | 0.1206 | 0.5205 | 1.0569 | 4.0788 | 3.6273 | | 4.7662 | 0.6148 | | 6.7593 |
| C0 | 0.0461 | 0.2305 | 0.1106 | 0.1587 | 0.1930 | | 0.3113 | 0.1689 | | 0.3649 |
| C2 | 0.0881 | 0.2614 | 0.2447 | 0.6385 | 0.0981 | | 0.5435 | 0.2465 | | 0.8327 |
| C3 | 0.0045 | 0.0106 | 0.0215 | 0.0510 | 0.0203 | | 0.1014 | 0.0170 | | 0.0858 |
| C4 | 0.0001 | 0.0019 | 0.0032 | 0.0091 | 0.0066 | | 0.0109 | 0.0024 | | 0.0238 |
| C6 | 0.0004 | 0.0026 | 0.0078 | 0.0163 | 0.0109 | | 0.0322 | 0.0163 | | 0.0175 |
| C8 | 0.0012 | 0.0015 | 0.0020 | 0.0126 | 0.0012 | | 0.0087 | 0.0037 | | 0.0219 |
| C10 | 0.0009 | 0.0055 | 0.0000 | 0.0200 | 0.0178 | | 0.0190 | 0.0123 | | 0.0217 |
| C12 | 0.0002 | 0.0004 | 0.0112 | 0.0256 | 0.0290 | | 0.0327 | 0.0049 | | 0.0467 |
| C14 | 0.0001 | 0.0021 | 0.0040 | 0.0145 | 0.0159 | | 0.0251 | 0.0104 | | 0.0303 |
| C16 | 0.0011 | 0.0129 | 0.0065 | 0.1234 | 0.0926 | | 0.2684 | 0.0718 | | 0.4477 |

***** Ala: alanine; Val: valine; Cit: citrulline; Leu: leucine; Met: methionine; Phe: phenylalanine; Pro: proline; Tyr: tyrosine; Gly: glycine; C0: pure free carnitine; C2: acetylcarnitine; C4: butyl carnitine; C8: octyl carnitine; C10: kwai carnitine; C3: propiylcarnitine; C6: hexanoylcarinitine; C12: lauroyl carnitine; C14: myristoyl carnitine; C16: palmitoyl carnitine.
